# Obsessive–compulsive and catatonic symptoms in the early stages of psychosis: Are they related?

**DOI:** 10.1007/s00406-025-02080-2

**Published:** 2025-10-06

**Authors:** Sergio Sanchez-Alonso, Manuel Canal-Rivero, Nathalia Garrido-Torres, Enrique Baca-Garcia, Benedicto Crespo-Facorro, Maria Luisa Barrigon, Miguel Ruiz-Veguilla

**Affiliations:** 1 Department of Psychiatry, Jimenez Diaz Foundation Hospital, Madrid, Spain; 2 Department of Health Sciences, Complutense University, Madrid, Spain; 3 Department of Psychiatry, Virgen del Rocío University Hospital, Seville, Spain; 4 Department of Psychiatry, Faculty of Medicine, University of Sevilla, Seville, Spain; 5 Seville Biomedical Research Centre (IBIS-CSIC), Seville, Spain; 6 Spanish Network for Research in Mental Health (CIBERSAM), Health Institute, Carlos III, Madrid, Spain; 7 Department of Psychiatry, UUniversity Hospital Fundación Jimenez Diaz, Talca, Chile; 8 Department of Psychiatry, University Hospital Rey Juan Carlos, Móstoles, Madrid, Spain; 9 Department of Psychiatry, General Hospital of Villalba, Madrid, Spain; 10 Department of Psychiatry, University Hospital Infanta Elena, Valdemoro, Madrid, Spain; 11 Department of Psychiatry, Universidad Católica del Maule, Talca, Chile; 12 Department of Psychiatry, Universidad Autónoma de Madrid, Madrid, Spain; 13 Institute of Psychiatry and Mental Health, Hospital GeneralUniversitario Gregorio Marañón, IiSGM School of Medicine,Complutense University, Madrid, Spain

**Keywords:** Catatonia, Obsessive–compulsive symptoms, Psychosis, Early-stage psychosis, First-episode psychosis

## Abstract

**Objective:**

Catatonia and obsessive–compulsive symptoms (OCS) are frequently observed in patients with psychosis, even at early stages, yet their relationship remains unclear. This study aimed to examine the prevalence and association of catatonic symptoms and OCS in early-stage psychosis.

**Methods:**

Seventy patients aged 18–55 years with early-stage psychosis (illness duration < 5 years) were assessed within 72 h of admission. Catatonic symptoms were evaluated using the Bush Francis Catatonia Rating Scale (BFCRS), OCS with the Obsessive–Compulsive Inventory-Revised (OCI-R), and psychotic symptoms with the Positive and Negative Syndrome Scale (PANSS). Correlations between catatonic, obsessive–compulsive, and psychotic symptom dimensions were analyzed. Logistic regression assessed associations between symptom dimensions and catatonia.

**Results:**

Catatonic symptoms (BFCRS ≥ 3) were present in 64.3% of patients, and 40% scored above the OCI-R cut-off for clinically significant OCS. Patients with catatonia had significantly higher total OCI-R scores and higher scores across all OCI-R subscales. Strong correlations were observed between mental neutralization and washing OCI-R subscales and BFCRS scores. Logistic regression showed that higher disorganized (OR = 1.699, 95% CI: 1.141–2.529, p = 0.009) and obsessive–compulsive symptoms (OR = 1.253, 95% CI: 1.058–1.483, p = 0.009) were independently associated with increased odds of catatonia, while positive symptoms showed a significant negative association (OR = 0.796, 95% CI: 0.644–0.983, p = 0.034).

**Conclusions:**

There is a high prevalence of both catatonic and obsessive–compulsive symptoms in early-stage psychosis. Disorganized and obsessive–compulsive symptoms are independent risk factors for catatonia, while positive symptoms may be protective. These findings highlight the importance of comprehensive symptom assessment in this population.

**Supplementary Information:**

The online version contains supplementary material available at 10.1007/s00406-025-02080-2.

## Introduction

Catatonia has been known since Kahlbaum first described catatonic syndrome in 1874 as a set of motor abnormalities, including symptoms such as mutism, negativism, stereotypies, catalepsy and verbigeration [1]. Although catatonia has traditionally been related to schizophrenia, its presence in other conditions, such as major affective disorders, has led to its recognition in the DSM 5 as a syndrome or specifier associated with other mental disorders [2], being approached in a transdiagnostic way by some authors [3].

Catatonia can be found in different psychiatric disorders, such as psychosis, mood disorders or neurodevelopmental disorders, as well as in different medical pathologies or in relation to the use of drugs [4]. Although often underdiagnosed, the prevalence of catatonia, regardless of the cause, is estimated to be 9.2% [4]. The highest percentages are observed in organic disorders and affective psychoses, whereas catatonic symptoms are present in around 10% of patients with schizophrenia [5].

Although the risk of worsening of catatonic symptoms with the use of antipsychotics is known [6], improvement of catatonic symptomatology with the use of antipsychotics has also been described [7]. Catatonia has been observed in drug-naive first-episode psychosis (FEP) patients [8, 9], and these symptoms may even precede the onset of psychosis [10].

The link between schizophrenia and OCD has been studied since classical psychiatry, explored by authors such as Westphal or Bleuler [11]. Although the presence of obsessive–compulsive symptoms in schizophrenia was initially considered to be associated with a better prognosis, subsequent research has demonstrated that these symptoms are linked to a potentially worse outcome of schizophrenia [12]. OCD has been described in 8 to 23% of patients with schizophrenia [13]. This comorbidity is highly prevalent even in pre-psychotic stages [14] and in drug-naïve FEP patients, affecting up 30% of patients [15]. Obsessive–compulsive symptoms (OCS) are present in 30–60% of patients with schizophrenia and in up to 37% of FEP patients [16].

Some studies report the prevalence of psychotic symptomatology in OCD to be between 39 and 44% of patients [17, 18]. On the other hand, the percentage of individuals with OCD who develop schizophrenia varies from 1 to 12% [19]. The incidence rate of schizophrenia is 10.93 times higher in patients with OCD compared to healthy controls (0.46/100 person-year Vs 0.04/100 person-year), and the risk of developing schizophrenia in patients with OCD reaches a hazard ratio of 10.46, with a higher risk in younger patients [20]. Importantly, the presence of psychotic disorders in OCD patients has been associated with impaired insight and functional deficits compared with OCD alone and should be considered a separate clinical entity [21].

Although motor disturbances and neurological soft signs (NSS) occur in Obsessive Compulsive Disorder (OCD) [22], catatonia is a scarcely documented phenomenon, with only a few case reports available [23–26].

Whereas certain antipsychotics can produce abnormal movements and catatonic symptoms, some antipsychotics have been associated with increased obsessive OCS, especially clozapine [27]. Even so, the high prevalence of OCS in drug-naïve FEP patients suggests a link between both disorders, regardless of antipsychotic treatment [8, 9].

Considering the potential relationship between schizophrenia and OCD, and the possibility of elevated motor symptoms, including catatonic symptoms, in individuals with both conditions, the present study aims to describe the presence of catatonic and obsessive–compulsive symptoms in a sample of patients in the early phases of psychosis, with a particular emphasis on patients experiencing their first episode of psychosis (FEP). Additionally, we intend to assess three symptomatic dimensions: psychotic, obsessive–compulsive and catatonic symptomatology, in this population and to identify their interrelationships. Specifically, we will study: 1) Differences in catatonic, obsessive–compulsive, and psychotic symptoms according to duration of psychosis (patients on their FEP and rest of patients) and specific diagnosis (affective and non-affective psychosis); 2) Correlations between catatonia, obsessive–compulsive symptoms, and psychotic domains, 3) Obsessive and psychotic symptoms in those patients who screened positive for catatonia and those who did not.

## Methods

### Participants

The sample comprised 70 patients with early-stage psychosis (less than 5 years of disease evolution) [28] admitted to the Mental Health Hospitalization Unit of the Virgen del Rocío University Hospital in Seville, Spain. All participants were assessed within 72 h of admission.

Exclusion criteria were (a) history of traumatic brain injury resulting in a loss of consciousness lasting one hour or more; and (b) history of neurological disease or any other condition capable of causing neurological abnormalities. The study was approved by the Local Clinical Research Ethics Committee of the Hospital Universitario Virgen del Rocío. Sevilla (Spain) (decision number PI-1378-N-18). Prior to participation in the study, all participants submitted and signed an informed consent form following a comprehensive explanation of the study procedure.

Among the sample, 54 patients (77%) were in their FEP and had never received antipsychotic medication at the time of admission, while 16 (33%) had their first psychotic episode in the previous five years (P5Y) and were taking antipsychotic medication. Regarding diagnosis, 49 (70%) were diagnosed with non-affective psychosis (46 with schizophrenia and four with brief psychotic episodes), and 21 (30%) were diagnosed with affective psychosis (manic episodes featuring psychotic symptoms).

### Assessment

Sociodemographic data including sex, age, and educational level, as well as clinical data were obtained from the patients and their relatives. Electronic health records were also used to retrieve information.

Catatonic symptoms were assessed using the Bush-Francis Catatonia Rating Scale (BFCRS) [29]. BFCRS consists of a 23-item rating scale. According a recent review, we consider a certain patient screened positive for catatonia when scored positive for three or more items [30], although BCRFS authors have previously proposed a cutoff point of 2 [29]. The remaining items are used to rate the severity of the symptoms: a score of 0 is assigned for the absence of a symptom; while the presence of a symptom is scored from 1 to 3, according to its clinical severity. Different authors have suggested that BFCRS can be divided into one to seven domains [31]. In this study, we adopted the three domains described by Subramaniyam et al. (retarded catatonia, excited catatonia and aberrant volitional catatonia) [31] to analyze the different dimensions of the patients’ catatoniform symptoms. The assessment was conducted by a board-certified psychiatrist from the Mental Health Hospitalization Unit (MRV).

Obsessive symptoms were assessed with the revised version of the Obsessive–Compulsive Inventory (OCI-R) [32]. OCI-R is composed of an 18-item rating scale, and each item is rated between 0 and 4 to assess the distress caused by the symptoms (0 = none; 1 = a little; 2 = a lot; 3 = very much; 4 = extreme). The OCI-R evaluates six symptom domains: hoarding, checking, ordering, mental neutralization, washing, and obsessing. While a total score of 21 or higher on the OCI-R indicates clinically significant OCS and may suggest possible OCD, it is important to clarify that the OCI-R is a screening tool and not a diagnostic instrument. In our study, we classified patients with a total OCI-R score greater than 21 as OCI-R ( +), indicating the likely presence of clinically significant OCS.

The Spanish version of the Positive and Negative Syndrome Scale (PANSS) [33] was used. PANSS is an instrument consisting of 30 items designed to assess positive symptoms (Items P1 to P7), negative symptoms (Items N1 to N7), and general psychopathology (Items G1 to G16) in patients with psychosis. For the purpose of our study, we used the five-factor model proposed by Marder to assess the different psychotic dimensions: negative symptoms, positive symptoms, disorganized thinking, hostility and anxiety/depression [34].

### Statistical analysis

Statistical analysis was conducted using SPSS, version 25. Kolgomorov-Smirnov test examined the normality of variables. Non-normally distributed variables were log-transformed. Catatonia and their subscales were normalized because they were skewed, as well as hoarding, checking, mental neutralization, washing, and obsessing OCI-R subscales; and the negative symptoms, hostility/uncontrolled arousal, and anxiety/depression PANSS’ Marder factors.

First, we described the sample using percentages, mean values, and standard deviation (M ± SD) for scales with a normal distribution and median and interquartile range (Mdn (IQR)) when the scale had a non-normal distribution.

In second place, we compared the three symptoms’ categories (catatonic, obsessive and psychotic) according to time from psychosis onset (FEP vs P5Y), and to diagnosis (non-affective psychosis versus affective psychosis).

Thirdly, we performed a correlation analysis between the three different domains of the BFCRS scale and OCI-R subscales and the five Marder’s factors of the PANSS. We tested the association or obsessive, catatonic, and psychotic symptoms with a correlation analysis with Pearson or Spearman test when corresponding. We considered correlations of 0–0.19 as very weak, 0.2–0.39 as weak, 0.40–0.59 as moderate, 0.6–0.79 as strong and 0.8–1 as very strong correlation [35].

Finally, we performed a logistic regression analysis incorporating the five PANSS-Marder factors to adjust for their potential influence on the relationship between obssesive-compulsive symptoms and catatonia*. *Using the Enter method, total scores of PANSS factors and OCIR were simultaneously included in the model.

## Results

### Sample description

A total of 70 patients aged 18–55 (mean = 32.6, SD = 10.68) composed the sample. Most of patients were in their FEP and suffered from a non-affective psychosis. Regarding symptomatology, BFCSR scores ranged 0–56 (median = 8, IQR = 16), with 64.3% of the sample scoring ≥ 3 on the BFCSI and fulfilling criteria of catatonia. The mean OCI-R score was 9.68 ± 12.51, and 40% of the sample achieved or surpassed the 21-point cut-off. The mean score of PANSS was 73.70 ± 19.23 (Table 1).

**Table 1:** Sociodemographic and clinical characteristics of total sample

	Total sample, n = 70
Age: years, mean ± SD; range	32.6 ± 10.68; 18–55
Gender male, n; %	35; 50%
Civil Status, n; %	
Single	45; 67.2%
Married	15; 22.4%
Separated	4; 5.7%
Widow	2; 2.9%
Divorced	1; 4.3%
Educational level, n; %	
Less than primary education	18; 27.3%
Primary education	24; 36.4%
Secondary education	14; 21.2%
University studies	10; 15.2%
Diagnosis: non-affective psychosis, n; %	49; 70%
FEP patients, n; %	57; 81.4%
BFCRS	
Retarded catatonia, Mdn; IQR	3.0; 8.00
Excited catatonia, Mdn; IQR	3.0; 5.50
Aberrant volitional catatonia, Mdn; IQR	1.0; 3.00
BFCRS total score, Mdn; IQR	8.0; 16.00
BFCSI Present items, Mdn; IQR	4.0; 5.25
BFCSI positive screening: ≥ 3 items positive	45; 64.3%
BFCSI positive screening: ≥ 2 items positive	51; 72.9%
OCI-R	
Hoarding, Mdn; IQR	3.0; 4.00
Checking, Mdn; IQR	3.0; 4.75
Ordering, mean ± SD	4.67 ± 2.98
Mental neutralization, Mdn; IQR	1.0; 3.00
Washing, Mdn; IQR	1.0; 2.00
Obsessing, Mdn; IQR	4.0; 6.00
OCI-R total score, mean ± SD	19.68 ± 12.51
OCI-R + : Cut-off ≥ 21	28; 40%
PANSS	
Positive factor, mean ± SD	73.70 ± 19.23
Negative factor, Mdn; IQR	12.5; 14.0
Disorganized thinking, mean ± SD	15.69 ± 6.53
Hostility, Mdn; IQR	7.0; 8.0
Anxiety/depression, Mdn; IQR	8.0; 5.5
PANS total score, mean ± SD	73.70 ± 19.23

According Oldham criteria (BFCSI ≥ 3) [30], in our sample 45 patients (64.3%) screened positive for catatonia, whereas median score for BFCSR was 8.0 (IQR 16.00). Within the catatonia domains, the most frequent was retarded catatonia. Among the specific catatonic symptoms measured, inmobility/stupor and staring occurred in more than the half of the sample (Supplementary Table s1).

The average score of OCI-R scale was 19,68 ± 12.51. Although the mean score was below the cut-off point, 28 patients (40%) reached or surpassed it, suggesting a comorbid OCD. The most frequent obsessive–compulsive dimension in our sample were ordering and obsessing. Items number 2, 3, 6 and 9 are highly prevalent and appears in more than the 70% of the patients. (Supplementary Table s2).

### Symptoms comparison between FEP patients and P5y patients

Patients with FEP scored statistically significantly than P5Ys on total BFCRS and excited and aberrant volitional domains. FEP patients also had more positive items on the BFCSI. Mental neutralization and washing domains of the OCI-R also scored higher on FEP patients. No differences were found for PANSS (Table 2).

**Table 2:** Differences between FEP and P5Y

Scales	FEP n = 57	P5Y n = 13	Statistics		
			t	df	p-value
BFCRS					
Retarded catatonia*	0.074 ± 1.06	− 0.32 ± 0.41	1.87	32.95	0.070
Excited catatonia*	**0.11 ± 1.06**	**− 0.49 ± 0.43**	**3.25**	**47.48**	**0.002**
Aberrant volitional catatonia*	**0.10 ± 1.07**	**− 0.46 ± 0.18**	**3.72**	**66.26**	** < 0.001**
Total BFCRS*	**0.11 ± 1.06**	**− 0.48 ± 0.37**	**3.42**	**56.33**	**0.001**
BFCSI Present items	**4.56 ± 3.56**	**3.00 ± 2.16**	**2.04**	**29.08**	**0.05**
OCI-R					
Hoarding*	0.089 ± 1.04	− 0.391 ± 0.68	1.58	68	0.118
Checking*	− 0.043 ± 0.93	0.191 ± 1.27	− 0.76	68	0.449
Ordering	4.543 ± 2.77	5.231 ± **3.85**	− 0.75	68	0.458
Mental neutralization*	**0.099 ± 1.05**	**− 0.437 ± 0.54**	**2.63**	**36.52**	**0.012**
Washing*	**0.086 ± 1.07**	**− 0.377 ± 0.45**	**2.44**	**45.94**	**0.018**
Obsessing*	0.013 ± 0.95	− 0.060 ± 1.23	0.23	68	0.812
Total OCI-R	19.56 ± 11.59	20.23 ± 16.53	− 1.38	14.81	0.892
PANSS					
Positive factor	24.45 ± 6.09	22.15 ± 7.94	1.14	64	0.25
Negative factor*	15.55	18.92	− 1.25	67	0.21
Disorganized factor	15.80 ± 6.27	15.23 ± 7.80	0.28	66	0.78
Hostility*	9.32	7.15	1.31	66	0.19
Anxiety/depression*	9.41	7.53	1.45	66	0.15
Total PANSS	74.39 ± 18.87	71.00 ± 21.19	0.56	62	0.57

^*^These variables were log-transformed

Statistically significant data are shown in bold

### Symptoms comparison between affective and non-affective psychosis

In patients with a non-affective psychosis, differences were found for PANSS and excited catatonia domain, but not for OCI-R (Table 3).

**Table 3:** Differences between non-affective psychosis and affective psychosis

Scales	Non-affective psychosis, n = 49	Affective psychosis, n = 21	Statistics		
			t	df	p-value
BFCRS					
Retarded catatonia*	− 0.05 ± 0.94	0.12 ± 1.12	− 0.68	68	0.498
Excited catatonia*	**− 0.15 ± 0.94**	**0.37 ± 1.05**	**− 2.09**	**68**	**0.040**
Aberrant volitional catatonia*	− 0.12 ± 0.83	0.28 ± 1.28	− 1.57	68	0.119
Total BFCRS*	− 0.13 ± 0.93	0.30 ± 1.12	1.66	68	0.102
BFCSI Present items	4.06 ± 3.41	4.76 ± 3.37	− 0.79	38.23	0.432
OCI-R					
Hoarding*	− 0.84 ± 0.88	0.20 ± 1.24	− 0.94	29.72	0.353
Checking*	− 0.01 ± 1.01	0.03 ± 1.00	− 1.51	38.12	0.881
Ordering	4.59 ± 3.11	4.86 ± 2.73	− 0.33	68	0.73
Mental neutralization*	− 0.12 ± 0.95	0.27 ± 1.08	− 1.49	68	0.139
Washing*	− 0.07 ± 0.93	0.16 ± 1.14	− 0.89	68	0.372
Obsessing*	− 0.01 ± 1.03	0.03 ± 0.94	− 0.18	68	0.854
Total OCI-R	19.34 ± 12.83	20.47 ± 12.02	− 0.34	68	0.732
PANSS					
Positive factor	24.11 ± 6.27	23.76 ± 7.11	0.20	64	0.841
Negative factor*	**0.24 ± 1.03**	**− 0.54 ± 0.70**	**3.63**	**54.82**	**0.001**
Disorganized factor	15.19 ± 6.64	11.23 ± 6.27	− 0.98	66	0.328
Hostility*	**− 0.19 ± 0.94**	**0.43 ± 1.01**	**− 2.47**	**66**	**0.016**
Anxiety/depression*	**− 1.17 ± 0.84**	**0.37 ± 1.23**	**− 2.14**	**66**	**0.035**
PANSS total	73.**86 ± 19.49**	73.35 ± 19.16	0.098	62	0.922

^*^These variables were log-transformed

Statistically significant data are shown in bold

### Correlations between catatonia, OCI-R and PANSS domains

We found strong to very strong and moderate correlations between mental neutralization and washing domains of OCI-R scales and BFCSR and subscales (Table 4). Moderate correlations were found between total PANSS score and Marder's disorganized factor score and the BFCRS and all domains (Table 4). Regarding OCI-R and PANSS, we only found strong correlations between Hostility Factor and Mental neutralization (R = 0.660; p < 0.0001).

**Table 4:** Correlation between catatonia and OCI-R and PANSS

	Total OCI-R	Hoarding	Checking	Ordering	Mental neutralization	Washing	Obsessing	PANSS total score	Positive	Negative	Disorganized	Hostility	Anxiety/ depression
Total BFCRS	0.203	0.234	0.119	0.185	0.851**	0.863**	0.135	0.6673**	0.347**	0.193	0.689**	0.413**	0.517**
Retarded catatonia	0.220	0.197	0.144	0.140	0.824**	0.910**	0.141	0.706**	0.313**	0.447**	0.654**	0.157	0.570**
Excited catatonia	0.192	0.217	0.122	0.194	0.818**	0.680**	0.096	0.534**	0.307**	0.009	0.623**	0.538**	0.316**
Aberrant volitional catatonia	0.081	0.190	0.011	0.139	0.577**	0.651**	0.095	0.486**	0.275**	-0.016	0.512**	0.406**	0.457**

^**^p < 0.0001; * P < 0.05

Statistically significant data are shown in bold

### Differences related to positive screening for catatonia.

When we compared scores of OCI-R and PANSS according being positive or negative for catatonia screening (BFCSI ≥ 3 items), we found higher scores in patients who screened positive for catatonia, with statistically significantly differences in both the OCI-R and all domains. Significantly higher scores were also observed for total PANSS and in the negative, disorganized and anxiety/depression factors (Table 5).

**Table 5:** Differences in OCI-R and PANSS according to catatonia screening

Scales			Statistics		
	BFCSI ≥ 3 items n = 45	BFCSI < 3 items n = 25	t	df	p-value
OCI-R					
Hoarding*	**3.64**	**1.88**	**3.23**	**66.31**	**0.002**
Checking*	**3.97**	**2.48**	**2.10**	**68**	**0.039**
Ordering	**5.28 ± 2.74**	**3.56 ± 3.14**	**2.40**	**68**	**0.019**
Mental neutralization*	**3.00**	**0.20**	**7.98**	**48.90**	** < 0.005**
Washing*	**2.20**	**0.00**	**6.43**	**44.00**	** < 0.005**
Obsessing*	**4.84**	**3.24**	**2.03**	**68**	**0.045**
OCI-R total	**22.98 ± 12.82**	**13.76 ± 9.61**	**3.13**	**68**	**0.003**
PANSS					
Positive factor	24.83 ± 6.14	22.43 ± 6.97	1.44	64	0.150
Negative factor*	**0.26 ± 1.07**	**− 0.50 ± 0.61**	**3.76**	**66.57**	** < 0.005**
Disorganized factor	**18.43 ± 5.91**	**10.66 ± 4.27**	**5.67**	**66**	** < 0.005**
Hostility*	0.11 ± 1.08	− 0.21 ± 0.80	1.24	66	0.210
Anxiety/depression*	**0.25 ± 1.12**	**− 0.46 ± 0.47**	**3.62**	**63.10**	**0.001**
PANSS Total	**81.00 ± 18.38**	**59.77 ± 11.85**	**5.58**	**59.16**	** < 0.005**

^*^These variables were log-transformed

Statistically significant data are shown in bold

The logistic regression analysis identified several significant associations between symptom dimensions and the presence of catatonic symptoms. Higher scores on the disorganized factor were significantly associated with increased odds of catatonia (OR = 1.699, 95% CI: 1.141–2.529, p = 0.009). Obsessive–compulsive symptoms (OCIR_total) were also significantly associated with an increased likelihood of catatonia (OR = 1.253, 95% CI: 1.058–1.483, p = 0.009). Conversely, positive symptoms showed a significant negative association with catatonia (OR = 0.796, 95% CI: 0.644–0.983, p = 0.034), indicating a potential protective effect. Negative symptoms demonstrated a non-significant trend toward increased risk (OR = 1.265, 95% CI: 0.976–1.640, p = 0.075). The hostility/excitement and anxiety/depression factors were not significantly associated with catatonia (Table 6).

**Table 6:** Logistic regression analysis of factors associated with catatonia

Predictor	B	SE	Wald	df	p-value	OR	95% C.I
OCIR total	**0.225**	**0.086**	**6.853**	**1**	**0.009**	**1.253**	**1.058–1.483**
PANSS Marder positive	**− 0.228**	**0.108**	**4.482**	**1**	**0.034**	**0.796**	**0.644–0.983**
PANSS Marder negative	0.235	0.132	3.160	1	0.075	1.265	0.976–1.640
PANSS Marder disorganized	**0.530**	**0.203**	**6.823**	**1**	**0.009**	**1.699**	**1.141–2.529**
PANSS Marder Hostility/excitement	0.123	0.165	0.563	1	0.453	1.131	0.819–1.562
PANSS Marder Anxiety/depression	0.330	0.207	2.543	1	0.111	1.391	0.927–2.088
Constant	**-10.520**	**4.114**	**6.537**	**1**	**0.011**	**0.000**	–

Statistically significant data are shown in bold

## Discussion

In this study, we examined the presence of catatonic and obsessive–compulsive symptoms in a sample of patients at early stages of psychosis and explored their potential association. A notably high occurrence of catatonic symptoms was observed, particularly among individuals with a first episode of psychosis (FEP). Obsessive–compulsive symptoms were also highly prevalent, with increased scores in the neutralizing and washing domains. A significant correlation was found between the BFCRS total score and obsessive–compulsive symptoms (especially in the neutralizing and washing dimensions) as well as with the PANSS disorganized factor. Moreover, patients who screened positive for catatonia showed a higher risk of presenting clinically significant obsessive–compulsive symptomatology, although the role of PANSS factors should be taking into account. These findings warrant further investigation in larger and longitudinal samples to better understand the clinical and neurobiological implications of these associations.

We observed a notably high prevalence of catatonia in our sample, with 64.3% of patients meeting criteria for catatonia when considering the presence of three or more items on the BFCSI [30, 36]. When applying a more inclusive threshold of two or more positive items, as proposed by Bush et al. [29] the prevalence increased to 70%. These rates are considerably higher than those typically reported in the literature, which range from less than 10 to 40% [36–39], even in studies using the same scale [36, 37, 39]. Several factors may explain these discrepancies. First, our study focused exclusively on patients in the early stages of psychosis, many of whom were drug-naïve, whereas other studies often included more heterogeneous samples with a broader range of diagnoses, including organic, neurotic [36], anxiety and dissociative disorders [37]. In the study with the most comparable sample-drug-naive, non-affective FEP patients-the prevalence of catatonic symptoms differed markedly. Peralta et al. [38] reported less than 20% catatonia in their sample, compared to 64% in ours. These discrepancies are likely attributable to differences in the catatonia rating scales employed. The assessment of our patients within 72 h of admission, during the acute phase of psychosis, may also have contributed to the high prevalence, as catatonic symptoms may overlap with the disorganized symptomatology of psychosis [40]. Furthermore, the use of dimensional assessment tools such as the BFCSI, and the application of a lower threshold for symptom count, likely increased the sensitivity for detecting milder or subthreshold catatonic signs that may be overlooked by stricter diagnostic criteria such as those of the DSM-5 [40].

The high percentage of catatonia found in our study suggests that the presence of catatonic symptoms in the early stages of psychosis before treatment is started is very common. Some works have studied catatonia in the early stages of psychosis, but this has been in the context of studying NSS rather than catatonia itself [38, 41–45].

Historically, there has been considerable debate regarding whether catatonia is more prevalent among patients with affective disorders or those with psychotic disorders [46]. Grover et al. found that the prevalence of catatonic symptomatology in affective psychoses is comparable to that in non-affective psychoses [37], whereas other authors have reported a higher frequency of catatonia in affective disorders [46]. In our sample, patients with affective psychosis were primarily diagnosed with psychotic mania. The literature indicates that catatonia is more frequently observed in patients with mania who exhibit greater illness severity, particularly those with prominent mixed features and more severe manic symptoms [47, 48]. Given the severity of the patients included in our study, this may have contributed to the higher scores observed in the excited catatonia domain.

Nevertheless, our findings support the notion that catatonic symptoms are frequently underdiagnosed in early psychosis [49]. Several factors may contribute to this underrecognition: catatonia is sometimes associated with more advanced stages of illness, and certain frequently occurring catatonic signs—such as staring or immobility/stupor—can be misinterpreted as typical features of psychosis. Despite this, the high frequency of catatonic symptoms observed in our sample highlights the potential value of systematic screening for catatonia in FEP as a means of improving early detection and clinical management. Interestingly, catatonic symptoms were more prevalent in patients with FEP compared to those with a longer duration of psychotic illness. One possible explanation is that FEP patients are generally naïve to antipsychotic treatment, which may have a therapeutic effect on catatonic symptoms. This possibility has been suggested by previous research [38], and further studies are needed to explore the impact of pharmacological interventions on catatonia in early psychosis.

We also observed a high prevalence of OCS in our sample, particularly within the neutralizing and washing domains. Notably, 40% of the patients scored above the clinical threshold (OCI-R > 21) on the OCI-R scale, indicating probable OCD. These rates are higher than those reported in previous studies [13, 15], where the prevalence of OCS in FEP populations typically ranges from 21 to 34%, depending on the assessment method used. The elevated rates observed in our study may be partly explained by the use of self-report instruments such as the OCI-R, which can capture subclinical symptoms, as well as by the fact that assessments were conducted during the acute phase of psychosis, when symptom severity is often heightened.

In our sample, the most frequently reported OCI-R dimensions were ordering and obsessing. This contrasts with previous findings, which have more commonly identified domains such as impaired control over mental activities, contamination, symmetry/tidiness, suspicion, and checking behaviors as the most prominent [16]. These discrepancies may be attributable to methodological differences, including the use of different assessment tools, as well as variations in diagnostic composition between samples; for instance, first-episode psychosis (FEP) in our study versus chronic schizophrenia in others.

Higher scores in the mental neutralization and washing domains of the OCI-R were observed among FEP in our sample. Although these specific OCS dimensions are not frequently highlighted in early psychosis literature, their prominence may reflect maladaptive coping strategies and cognitive biases present in this population [50]. Mental neutralization behaviors could arise as attempts to manage distressing thought disturbances commonly experienced during the onset of psychosis. It is also possible that the use of a self-administered instrument such as the OCI-R contributed to the higher reporting of these symptoms, as patients may interpret certain psychosis-related behaviors as obsessive–compulsive in nature.

The lack of significant differences in obsessive–compulsive symptoms (OCS) between affective and non-affective psychoses may be partly explained by characteristics of the sample. As the participants were in the early stages of psychosis, or in some cases experiencing a first episode of psychosis (FEP), it is possible that certain clinical features had not yet fully emerged or stabilized at the time of assessment. This temporal factor could have masked potential diagnostic distinctions. Nevertheless, the observed pattern supports the notion that OCS may represent a transdiagnostic phenomenon in early psychosis, warranting further exploration in longitudinal studies.

We observed a strong correlation between catatonic symptomatology and the obsessive–compulsive domains of neutralization and washing. This association aligns with prior clinical observations and may indicate that obsessive symptoms in these domains are accompanied by notable psychomotor abnormalities, manifesting as both hyperactivity and inhibition. Additionally, we found a moderate correlation between the BFCRS score and the disorganized factor of the PANSS, which is consistent with findings from earlier studies [8]. These results suggest that disorganization in psychosis may influence psychomotor function, potentially through disruptions in volition. However, given the cross-sectional nature of our study, causal inferences should be made with caution, and further research is warranted to clarify the direction and mechanisms underlying these associations.

We also observed that patients who screened positive for catatonia tended to have higher scores on the total OCI-R and across all of its subdomains, as well as elevated scores on the total PANSS and on its negative, disorganization, and anxiety/depression factors. These findings are consistent with previous research reporting frequent comorbidity between catatonic features and anxious or depressive symptoms. Nonetheless, given the observational design of the study, caution is warranted when interpreting these associations, as they may be influenced by shared underlying factors or assessment overlaps rather than direct causal relationships. [51].

Furthermore, it can be confirmed from this sample that the presence of obsessive–compulsive symptomatology (OCI-R scores above 21) acts as a risk factor for the development of catatonic symptomatology. Likewise, catatonic symptomatology also acts as a risk factor for obsessive symptomatology. This novel finding would be worth considering for future research, as it supports the relationship between obsessive and catatonic symptomatology in the early stages of psychosis.

The findings from the logistic regression analysis indicate that higher levels of psychotic disorganization and obsessive–compulsive symptoms are independent risk factors for catatonia, with odds ratios of 1.699 and 1.253, respectively. These results are consistent with previous studies linking cognitive disorganization to alterations in fronto-striatal networks and repetitive OCD behaviors to rigid motor patterns characteristic of catatonia [52, 53]. In contrast, positive symptoms were inversely associated with catatonia (OR = 0.796), suggesting differential neurobiological mechanisms; this may be explained by increased dopaminergic activation in positive psychoses, which could counteract the GABAergic hypoactivity typically seen in catatonia [54, 55]. Although negative symptoms showed only a non-significant trend toward increased risk (OR = 1.265), their direction aligns with studies reporting a higher prevalence of negative symptomatology in catatonic patients [56]. No significant associations were observed for hostility/excitement or anxiety/depression dimensions. These results should be interpreted with caution, as the cross-sectional nature of the data precludes causal inference. Further research is warranted to clarify the underlying mechanisms and confirm these associations in larger, prospective samples.

Our findings provide support for the potential shared mechanisms between psychosis and OCD which have previously been identified [57]. Both disorders share common clinical features involving NSS; when occurring comorbidly in either psychosis with OCD or OCD with psychotic symptoms, they display a worse prognosis and worse general functioning [21]. The peculiarities in the presentation of this comorbidity highlight a distinct phenotype within schizophrenia, which some authors call “schizo-obsessive disorder” [57]. We hypothesize that motor symptoms act as a link between psychosis and OCD, potentially sharing a common biological substrate.

### Strengths and limitations

Our work is original in investigating the link between obsessive symptoms and catatonia, an area where research has been sparse. One of the strengths of the study is the assessment of a sample of patients in the early stages of psychosis under real-world conditions. While the data obtained on the prevalence of obsessive symptoms are consistent with previous studies, the prevalence of catatonic symptoms is higher in our study, which may reflect an underdiagnosis of catatonia. However, certain limitations must be recognized, although the sample is homogeneous in terms of diagnoses, our assessment did not include data on substance use or other related factors, such as cognitive profiles or NSS. Furthermore, we did not register data on treatment during admission and at discharge. The assessment of our patients within 72 h of admission, during the acute phase of psychosis, may also have contributed to the high prevalence, as catatonic symptoms may overlap with the disorganized symptomatology of psychosis. Given that the study is conducted in a population in early stages of psychosis, it is unclear whether patients may present diagnostic instability. Therefore, a more prolonged study period could yield additional data on the link between obsessive, catatonic and psychotic symptoms. Larger samples and replication studies are necessary to verify the potential link between OCS and the development of catatonic symptoms.

Additional limitations include the relatively small sample size and the single-center nature of the study, which may limit generalizability. The cross-sectional design prevents the establishment of causal relationships between variables. It is important to note that we did not systematically assess or exclude comorbid psychiatric disorders beyond the primary psychotic diagnosis, which could potentially influence the results, particularly regarding obsessive–compulsive symptoms.

## Conclusions

We found a high prevalence of obsessive and catatonic symptoms in patients in the early stages of psychosis, particularly in FEP patients and a strong correlation between catatonic symptoms and obsessive symptoms, specifically mental neutralization and washing, and moderate correlation with psychotic disorganization. Although more research is needed, this finding suggests a possible link between motor symptoms, psychosis and OCS. Furthermore, we found a high presence of obsessive symptoms that seem to increase the risk of presenting catatonic symptomatology. This is also the case with elevated clinical disorganized presence. In light of our results, and given the clinical and prognostic implications, we suggest the systematic evaluation of catatonic symptomatology in the evaluation of patients with FEP.

## Supplementary Information

Below is the link to the electronic supplementary material.

Supplementary file1 (DOCX 19 KB)

## Data Availability

The dataset used and/or analyzed during the current study are available under reasonable request to the corresponding author.
