# Supplementary material for: Obsessive–compulsive and catatonic symptoms in the early stages of psychosis: Are they related?
Source: Eur Arch Psychiatry Clin Neurosci. 2025 Oct 6;275(8):2523–33. doi: 10.1007/s00406-025-02080-2 (PMC12638396; doi:10.1007/s00406-025-02080-2)
Supplement: Supplementary file 1 — Supplementary file1 (DOCX 19 KB) [file 406_2025_2080_MOESM1_ESM.docx]

**SUPLEMENTARY TABLES**

**Table s1: Percentage of positive catatonic symptoms**

| **BFCSR Items** | **Present**  **N; %** | **Mdn; IQR** |
| --- | --- | --- |
| 1. **Excitement** | 32; 45.7% | 0 .0; 2.0 |
| 1. **Immobility/Stupor** | **36; 51.4%** | 1.0; 2.0 |
| 1. **Mutism** | 53; 32.9% | 0.0; 1.0 |
| 1. **Staring** | **36; 51.4%** | 1.0; 2.0 |
| 1. **Posturing‎/Catalepsy** | 32; 45.7% | 0.0; 2.0 |
| 1. **Grimacing** | 26; 37.1% | 0.0; 1.0 |
| 1. **Echopraxia‎/Echolalia** | 7; 10% | 0.0; 0.0 |
| 1. **Stereotypy** | 19; 27.1% | 0.0; 1.0 |
| 1. **Mannerisms** | 53; 32.9% | 0.0; 1.0 |
| 1. **Verbigeration** | 22; 31.4% | 0.0; 1.0 |
| 1. **Rigidity** | 8; 11.4% | 0.0; 0.0 |
| 1. **Negativism** | 17; 24.3% | 0.0; 1.0 |
| 1. **Waxy flexibility** | 3; 4.3% | 0.0; 0.0 |
| 1. **Withdrawal** | 14; 20% | 0.0; 0.0 |
| 1. **Impulsivity** | 21; 30% | 0.0; 1.0 |
| 1. **Automatic obedience** | 17; 24.3% | 0.0; 1.0 |
| 1. **Mitgehen** | 10; 14.3% | 0.0; 0.0 |
| 1. **Gegenhalten** | 5; 7.1% | 0.0; 0.0 |
| 1. **Ambitendency** | 18; 25.7% | 0.0; 3.0 |
| 1. **Grasp Reflex** | 1; 1.4% | 0.0; 0.0 |
| 1. **Perseveration** | 10; 14.3% | 0.0; 0.0 |
| 1. **Combativeness** | 17; 24.3% | 0.0; 1.0 |
| 1. **Autonomic Abnormality** | 32; 45.7% | 0.0; 2.0 |

**Table s2: Percentage of positive obsessive-compulsive symptoms**

| **OCI-R Items** | **Present**  **N; %** | **Mdn; IQR** |
| --- | --- | --- |
| 1. **I have saved up so many things that they get in the way.** | 41; 58.6% | 1.0; 2.0 |
| 1. **I check things more often than necessary.** | **51; 72.9%** | 1.0; 2.0 |
| 1. **I get upset if objects are not arranged properly.** | **57; 81.4%** | 2.0; 2.0 |
| 1. **I feel compelled to count while I am doing things.** | 33; 47.1% | 0.0; 1.0 |
| 1. **I find it difficult to touch an object when I know it has been touched by strangers or certain people.** | 26; 37.1% | 0,0; 1.0 |
| 1. **I find it difficult to control my own thoughts.** | **51; 72.9%** | 1.0; 3.0 |
| 1. **I collect things I don’t need.** | 38; 54.3% | 1.0; 2.0 |
| 1. **I repeatedly check doors, windows, drawers, etc.** | 36; 51.4% | 1.0; 1.0 |
| 1. **I get upset if others change the way I have arranged things.** | **50; 71.4%** | 1.0; 3.0 |
| 1. **I feel I have to repeat certain numbers.** | 24; 34.3% | 0.0; 1.0 |
| 1. **I sometimes have to wash or clean myself simply because I feel contaminated.** | 26; 37.1% | 0.0; 1.0 |
| 1. **I am upset by unpleasant thoughts that come into my mind against my will.** | 48; 68.6% | 1.0; 2.0 |
| 1. **I avoid throwing things away because I am afraid I might need them later.** | 38; 54.3% | 1.0; 1.0 |
| 1. **I repeatedly check gas and water taps and light switches after turning them off.** | 33; 47.1% | 0.0; 2.0 |
| 1. **I need things to be arranged in a particular way.** | 39; 55.7% | 1.0; 2.0 |
| 1. **I feel that there are good and bad numbers.** | 25; 35.7% | 0.0; 1.0 |
| 1. **I wash my hands more often and longer than necessary.** | 30; 42.9% | 0.0; 1.0 |
| 1. **I frequently get nasty thoughts and have difficulty in getting rid of them.** | 40; 57.1% | 1.0; 2.0 |
